# Endoscopic Management of Pancreaticobiliary Injuries: A Level 1 US Trauma Center Experience

**DOI:** 10.31486/toj.24.0040

**Published:** 2024

**Authors:** Spencer R. Goble, Mohamed Abdallah, Carly Rosenberg, Ahmed Dirweesh, Robert Matlock

**Affiliations:** ^1^ Department of Internal Medicine, Hennepin Healthcare, Minneapolis, MN; ^2^ Department of Gastroenterology, University of Minnesota Medical Center, Minneapolis, MN; ^3^ Department of Gastroenterology, University of Rochester, Rochester, NY; ^4^ Department of Gastroenterology, Hennepin Healthcare, Minneapolis, MN

**Keywords:** *Abdominal injuries*, *cholangiopancreatography–endoscopic retrograde*, *retrospective studies*, *wounds–gunshot*, *wounds–nonpenetrating*

## Abstract

**Background:** Traumatic pancreaticobiliary injuries are challenging to diagnose and manage. Endoscopic retrograde cholangiopancreatography (ERCP) has potential diagnostic and therapeutic utility in cases of traumatic pancreaticobiliary injuries.

**Methods:** In this single-center retrospective study, we assessed 25 cases of abdominal trauma in which the patients underwent ERCP for management of suspected pancreaticobiliary injuries. We analyzed basic patient demographics, mechanism of trauma, method of diagnosis, ERCP results, surgical treatments, and outcomes.

**Results:** Of the 25 assessed patients, 12 (48%) had pancreatic injuries, 12 (48%) had biliary injuries, and 1 (4%) patient had both. The median age was 28 years [IQR 25-35], and 84% of patients were males. Fifty-six percent of injuries were from blunt trauma, while 44% were from penetrating trauma. In cases of ERCP-confirmed biliary leaks (n=11), 100% of leaks were resolved in the 8 patients who underwent repeat ERCP after initial ERCP with stenting. In cases of ERCP-confirmed pancreatic duct leaks (n=10), 57% of duct leaks were resolved in the 7 patients who underwent repeat ERCP after initial ERCP with stenting. One patient in the biliary trauma cohort developed post-ERCP pancreatitis and sepsis.

**Conclusion:** ERCP was a useful diagnostic and therapeutic intervention in this population of patients with pancreaticobiliary trauma.

## INTRODUCTION

Traumatic pancreatic and biliary injuries present diagnostic and therapeutic dilemmas. The rare nature of the injuries and the nonspecific symptoms of each can result in the injuries easily being missed, particularly in the trauma setting when the focus may be on more readily apparent injuries.^1-5^ Additionally, computed tomography (CT) has a relatively low sensitivity for identifying pancreatic duct trauma and biliary leaks, further increasing the risk for delayed diagnosis.^1,2,6,7^ Magnetic resonance imaging may not be feasible in the trauma setting, when multiple injuries are present, and when metallic implants are present. Pancreatic duct leaks and biliary leaks are both associated with significant morbidity that increases with delays in diagnosis, emphasizing the importance of identifying them promptly in trauma patients.^3,4,8-11^ In cases of pancreatic trauma, the rate of significant morbidity doubles when the diagnosis is delayed.^4^

Endoscopic retrograde cholangiopancreatography (ERCP) is the gold standard for diagnosis of bile and pancreatic duct leaks.^1^ Additionally, ERCP is a potentially therapeutic intervention in these cases, with prior studies finding ERCP with stenting to be effective in controlling pancreatic duct and biliary leaks in approximately 80% and 90% of cases, respectively.^12-16^ While assessment of the utility of ERCP for pancreaticobiliary injuries specifically in the trauma setting is limited, available studies suggest that ERCP is safe and is an effective diagnostic and treatment option for this population.^1,4,8,12^ In this study, we examined the role of ERCP in diagnosis and management of pancreatic and biliary injuries in patients with blunt and penetrating trauma.

## METHODS

This retrospective analysis included all patients who underwent ERCP for suspected biliary or pancreatic injury after abdominal trauma from January 1, 2010, through September 30, 2020, at a Level 1 trauma center in a tertiary care institution. Cases of both blunt and penetrating abdominal trauma were included. Individuals with traumatic pancreaticobiliary injuries who did not undergo ERCP were excluded. We reviewed patient notes, laboratory results, imaging reports, operative reports, and ERCP reports and recorded basic demographics for each patient. We recorded the circumstances and initial presentation for each injury, with the severity of injury characterized by the Injury Severity Score (ISS). The ISS is calculated by assigning a numeric score that correlates with injury severity (0 to 5, ranging from no injury to unsurvivable injury) to 6 different body systems, squaring the 3 highest scores, and adding those scores for a final score of 0 to 75. We also reviewed and summarized the diagnostic workup for each patient, including imaging and laboratory results.

Patients were categorized by the management of their pancreaticobiliary trauma: surgical, endoscopic, percutaneous, and combinations of the different modalities. Procedural adverse events were assessed from procedural reports and daily progress notes. For assessments of management and outcomes, patients were categorized as having a pancreatic injury, biliary injury, or both. Patient demographics, diagnostics, management, and outcomes are all summarized using standard descriptive statistics. STATA version 17.0 (StataCorp LLC) was used for statistical computations.

The study was approved by the Hennepin Healthcare Institutional Review Board, and all study procedures were in accordance with the 1964 Declaration of Helsinki and its subsequent amendments.

## RESULTS

### Demographic and Clinical Characteristics

Twenty-five patients were evaluated: 12 patients (48%) had a traumatic pancreatic injury, 12 (48%) had a traumatic biliary injury, and 1 (4%) had both (Table 1). Age at presentation ranged from 14 to 60 years, with a median age of 28 years [IQR 25-35]; most of the cohort was male (84%) and White (64%). Blunt force injury was common in the pancreatic trauma cohort (8/12, 67%), while penetrating injury was common in the biliary trauma cohort (7/12, 58%). Gunshot was the most common mechanism of injury, accounting for 40% of presentations. Associated injuries in addition to pancreatic and biliary trauma were common (24/25, 96%). Intra-abdominal hematoma or active vessel bleeding was noted in 7 patients (28%), including 1 patient with an injury to the inferior vena cava and another with an injury to the superior mesenteric artery; both patients were managed surgically. Two patients (8%) required partial colectomy, 5 patients (20%) required splenectomy, and 3 patients (12%) required cholecystectomy. Ten patients (40%) had serious intrathoracic injuries, including pneumothorax and pulmonary contusion.

**Table 1.** Baseline Characteristics of Patients With Biliary/Pancreatic Duct Injuries Requiring Endoscopic Retrograde Cholangiopancreatography, n=25

Variable	Value
Age, years, median [IQR]	28 [25-35]
Male	21 (84)
Race/ethnicity	
Caucasian/White	16 (64)
African American/Black	5 (20)
Hispanic	2 (8)
Other	2 (8)
Mechanism of trauma	
Blunt	14 (56)
Penetrating	11(44)
Type of trauma	
Gunshot wound	10 (40)
Blunt object	5 (20)
Motor vehicle accident^a^	4 (16)
Penetrating trauma with a knife or sharp object	1 (4)
Fall from building	1 (4)
Go-cart accident	1 (4)
Snowmobile accident	1 (4)
Pedestrian hit by train	1 (4)
Crush injury	1 (4)
Circumstances of trauma	
Accidental	13 (52)
Assault	10 (40)
Work-related	1 (4)
Unknown	1 (4)
Type of injury	
Biliary	12 (48)
Pancreatic	12 (48)
Both	1 (4)
Injury Severity Score, median [IQR]	22 [16-34]
Exploratory laparotomy on presentation	17 (68)
Hepatobiliary/pancreatic surgery performed	7 (28)
Hypovolemic shock on presentation	7 (28)
Blood products administered^b^	
Packed red blood cells	17 (68)
Fresh frozen plasma	9 (36)
Total parenteral nutrition administered	9 (36)
ICU length of stay, days, median [IQR]	3 [1-10]
Hospital length of stay, days, median [IQR]	19 [13-26]

^a^The patient who had both biliary and pancreatic injuries sustained those injuries in a motor vehicle accident. The patient is included in both the biliary trauma cohort and the pancreatic trauma cohort in the Results section but is counted once here.

^b^One patient received both packed red blood cells and fresh frozen plasma.

Note: Data are presented as n (%) unless otherwise indicated.

ICU, intensive care unit; IQR, interquartile range.

### Biliary Trauma Cohort

The median age of the 13 patients who had documented concern for biliary injury was 28 years [IQR 24-43], and 85% (11/13) were male. Of the 7 cases of penetrating abdominal injury, 6 (86%) were due to gunshot wounds, with the remaining case due to a stab wound. Of the 6 cases of blunt force injuries, 2 (33%) were secondary to being struck with a blunt object, 2 (1 was the patient with both biliary and pancreatic trauma) resulted from motor vehicle accidents, while a snowmobile accident and being struck by a train accounted for the remaining 2 cases.

Concern for biliary injury was first established by CT as part of the initial trauma workup in 77% of patients (10/13), and the remaining 23% of patients (3/13) had concern first established during exploratory laparotomy done on initial presentation.

CT was performed prior to ERCP in 77% of patients (10/13), and hepatic lacerations were noted on all 10 CTs. In 5 of the 10 cases (50%), perihepatic fluid collections were seen on the CTs, and in all 5 of those cases (100%), biliary leak was later confirmed on ERCP. A bile leak was later confirmed in 3 of the 5 cases (60%) where initial CT showed no perihepatic fluid collection. One patient underwent magnetic resonance cholangiopancreatography (MRCP) prior to ERCP. The MRCP did not demonstrate any biliary leak, and none was noted on the ERCP.

Six of the 13 patients (46%) with documented concern for biliary injury had hepatobiliary iminodiacetic acid (HIDA) scans, and 5 of the HIDA scans (83%) documented bile leaks, all of which were later confirmed on ERCP. One HIDA scan was negative for any biliary leak; however, ERCP later demonstrated a bile leak in that patient.

ERCP was performed within 6 days of the initial biliary trauma in 31% of cases (4/13) and within 14 days in 69% of cases (9/13) (Table 2).

**Table 2.** Management and Outcomes of Biliary and Pancreatic Duct Injuries

Variable	Biliary Trauma, n=12	Pancreatic Trauma, n=12	Biliary and Pancreatic Trauma, n=1
Imaging performed prior to ERCP			
Computed tomography	9 (75)	12 (100)	1 (100)
Magnetic resonance cholangiopancreatography	1 (8)	4 (33)	0
Hepatobiliary iminodiacetic acid scan	5 (42)	0	1 (100)
Time from trauma to diagnosis of biliary or pancreatic trauma, days, median (range)	7 (0-196)	0 (0-20)	1
Management modality			
Endoscopic only	2 (17)	0	
Endoscopic and percutaneous	3 (25)	4 (33)	1 (100)
Endoscopic and surgical	1 (8)	0	
Endoscopic, percutaneous, and surgical	6 (50)	8 (67)	
Number of days from time of trauma to ERCP			
0-6	3 (25)	6 (50)	1 (100)
7-14	5 (42)	2 (17)	
>14	4 (33)	4 (33)	
Location of biliary leak^a^			
Common bile duct	1 (10)	N/A	1 (100)
Right hepatic duct	4 (40)	N/A	
Left hepatic duct	1 (10)	N/A	
Intrahepatic ducts	4 (40)	N/A	
Location of pancreatic leak^b^			
Head	N/A	5 (56)	1 (100)
Body	N/A	1 (11)	
Tail	N/A	3 (33)	
Octreotide use	0	5 (42)	0
Pseudocyst development	0	4 (33)	0
Number of percutaneous drainages, median (range)	2 (0-3)	1.5 (1-2)	1
Percutaneous drain in place, days, median [IQR]	29 [3-46]	42 [32-48]	40
Number of ERCPs performed, median (range)	2 (1-7)	2 (1-4)	5
Length of follow-up, days median [IQR]	456 [203-909]	128 [91-244]	139

^a^The number of bile leaks in the biliary trauma cohort = 10, while the total number of bile leaks (including the patient with biliary and pancreatic trauma) = 11.

^b^The number of pancreatic duct leaks in the pancreatic trauma cohort = 9, while the total number of pancreatic duct leaks (including the patient with biliary and pancreatic trauma) = 10.

Note: Data are presented as n (%) unless otherwise indicated.

ERCP, endoscopic retrograde cholangiopancreatography; IQR, interquartile range; N/A, not applicable.

**Figure 1.**
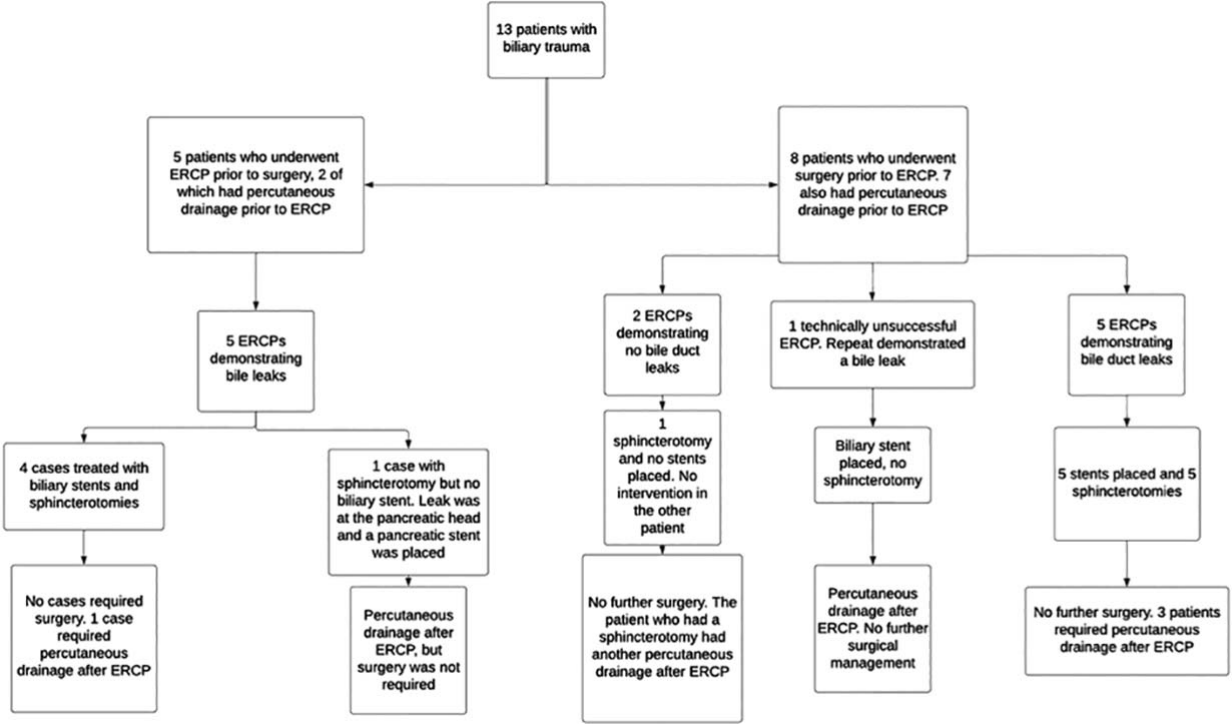
**Initial management of patients with traumatic biliary injury. One patient had both biliary and pancreatic injuries.** ERCP, endoscopic retrograde cholangiopancreatography.

In the 11 cases of ERCP-confirmed biliary leak, management included biliary stenting and sphincterotomy in 9 patients (82%), sphincterotomy alone in 1 patient (9%), and stenting without documentation of sphincterotomy in 1 patient (9%) (Figure 1).

Eight of the 11 patients with confirmed biliary leak underwent repeat ERCP, and resolution of the biliary leak was demonstrated in all 8 patients (100%). Repeat ERCP was recommended for the other 3 patients with a confirmed biliary leak, but they were lost to follow-up. Consequently, 73% of patients (8/11) with confirmed biliary leak had resolution confirmed by ERCP.

Eight of the 13 patients (62%) with biliary trauma underwent exploratory laparotomy prior to any endoscopic intervention and had ERCP performed after surgery (Figure 1). All 8 surgeries were performed to manage associated intra-abdominal injuries; biliary leaks were not repaired during these surgeries. Of these 8 patients, none required further surgeries after the ERCP. The other 5 patients (38%) had an ERCP without prior surgery, and none required subsequent surgery.

Of the 13 patients with a biliary injury, 9 (69%) required 2 or fewer ERCPs during the study period, and the median number of ERCPs in patients with only a biliary injury was 2 (Table 2). One patient developed sepsis and pancreatitis shortly after ERCP, but no other procedural adverse events were documented. Seven of the 13 patients (54%) eventually developed hepatic abscesses, 6 of whom (86%) required percutaneous drainage. Two patients developed infected bilomas. No deaths occurred in the biliary trauma cohort.

### Pancreatic Trauma Cohort

The median age of the 13 patients who had documented concern for pancreatic injury was 29 years [IQR 26-35], and 85% (11/13) were male. All 4 cases of penetrating abdominal injury were due to a gunshot wound. Of the 9 cases of blunt force injuries, 3 (33%) were secondary to being struck with a blunt object; 3 (1 was the patient with both biliary and pancreatic trauma) were due to motor vehicle accidents; and a fall from a building, a go-cart accident, and a machinery accident (crush injury) at work accounted for the remaining 3 cases.

Concern for pancreatic injury was first established by CT as part of the initial trauma workup in 92% of cases (12/13), and the remaining patient had concern first established during exploratory laparotomy.

Four of the 13 patients (31%) had peripancreatic fluid collections found on initial CT, and each patient had a pancreatic duct leak later confirmed on ERCP. Three of the 13 patients (23%) had pancreatic lacerations noted on CT, and each patient was found to have a pancreatic duct leak on ERCP. Three of the 13 patients (23%) had pancreatic transections on CT; however, only 2 of the 3 (67%) were found to have a duct leak on ERCP. Two of the 13 patients (15%) had peripancreatic hematomas on initial CT, but neither had a pancreatic duct leak on subsequent ERCP. The remaining patient had no signs of pancreatic trauma on CT but was later found to have a pancreatic duct leak on ERCP.

Four of the 13 patients (31%) had MRCPs prior to ERCP. One MRCP correctly identified a pancreatic duct disruption that was later confirmed on ERCP. Another MRCP visualized an intact duct that ERCP confirmed did not have a leak. The duct was not well visualized on the MRCP for the third patient, but ERCP later identified a leak in this patient. The MRCP for the fourth patient showed pancreatic transection, but no leak was found on subsequent ERCP.

ERCP was performed within 6 days of the initial pancreatic trauma in 54% of cases (7/13) and within 14 days in 69% of cases (9/13) (Table 2).

In the 10 cases of ERCP-confirmed pancreatic duct leak, 7 patients (70%) underwent repeat ERCP, and resolution of the duct leak was demonstrated in 4 of the 7 patients (57%). Among the remaining 3 patients who underwent repeat ERCP, 2 patients (67%) were lost to follow-up after the second ERCP. The third patient underwent a third ERCP, the duct leak was still present, and the patient was then lost to follow-up. Repeat ERCP was recommended for the other 3 of 10 patients with a confirmed pancreatic duct leak, but all 3 patients were lost to follow-up. In total, 40% of patients (4/10) with a confirmed pancreatic duct leak had resolution confirmed by ERCP.

**Figure 2.**
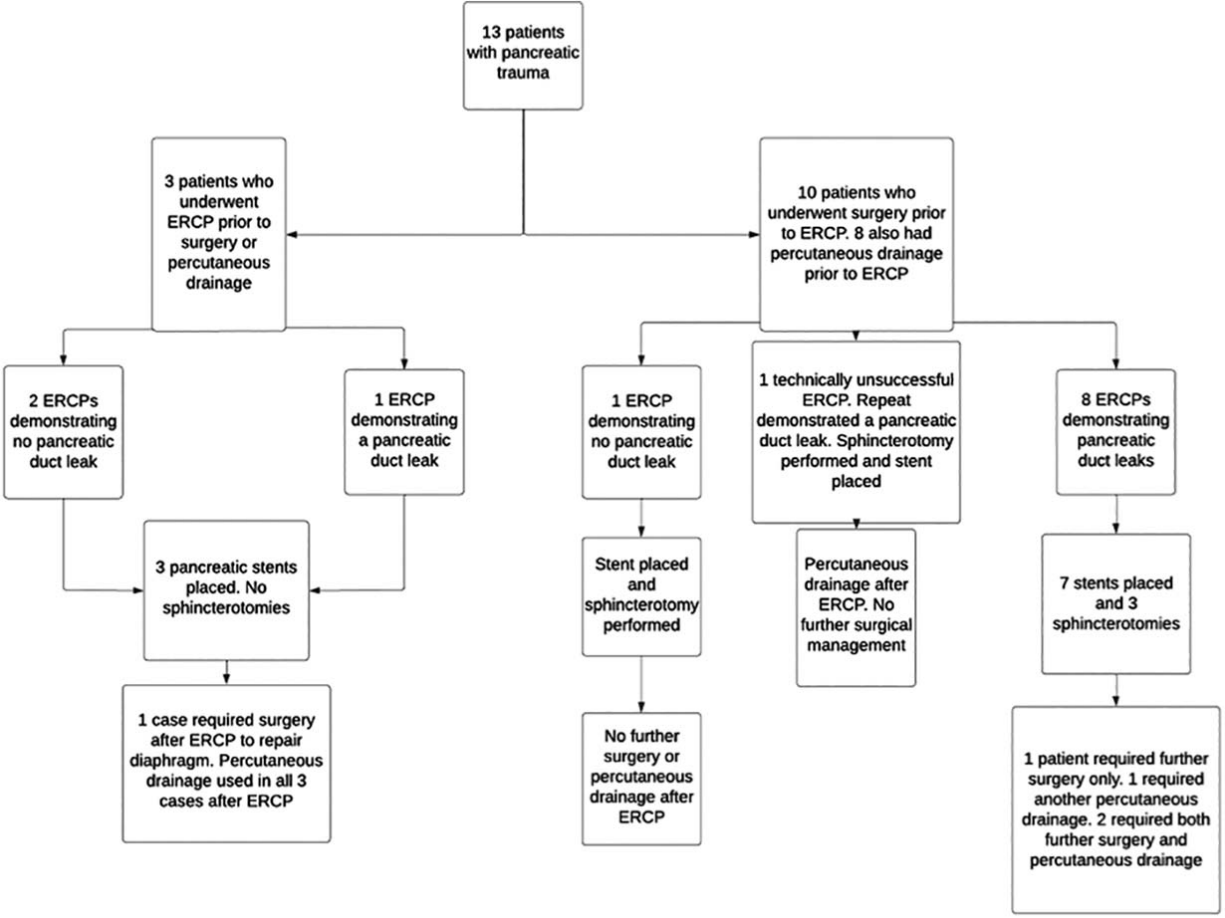
**Initial management of patients with traumatic pancreatic injury. One patient had both biliary and pancreatic injuries.** ERCP, endoscopic retrograde cholangiopancreatography.

Ten of the 13 patients (77%) with pancreatic trauma underwent surgery prior to any endoscopic intervention and had ERCP performed after surgery (Figure 2). Three patients had distal pancreatectomies during the initial exploratory laparotomy. All 3 patients were subsequently found to have distal pancreatic duct leaks on ERCP, but the patient notes did not specify if the leaks were secondary to the initial trauma or to surgical adverse events. Of the 10 patients with pancreatic injuries who underwent surgery prior to ERCP, 3 (30%) required repeat surgery after ERCP to manage intra-abdominal injuries, including 1 patient who required further pancreatic resection after previous pancreatic resection.

Three of the 13 patients (23%) with pancreatic trauma had an ERCP without prior surgery. One patient required surgical intervention after ERCP to repair a diaphragm injury, but no specific surgical procedure for the pancreatic trauma was needed. The other 2 patients were successfully managed with endoscopy and percutaneous drainage alone.

No ERCP-related adverse events were noted in the pancreatic cohort; however, 4 patients (31%) developed pancreatic pseudocysts, and 1 patient (8%) developed a peripancreatic abscess. Two of the patients with pancreatic pseudocysts required percutaneous drainage, while the other 2 patients were managed solely with endoscopic stenting of the pancreatic duct. The peripancreatic abscess was successfully managed with percutaneous drainage. No deaths occurred in the pancreatic trauma cohort.

## DISCUSSION

ERCP was well tolerated in this population of 25 patients, with adverse events (pancreatitis and sepsis) noted in only 1 patient (4%). ERCP also appeared to be effective, particularly for patients with biliary trauma, as 100% of biliary leaks were resolved on repeat ERCP after stenting (8 of 8 patients), and 57% of pancreatic duct leaks were resolved on repeat ERCP (4 of 7 patients).

Diagnosis of biliary injuries requires a high index of suspicion as routine trauma imaging with abdominal CT may not demonstrate the injury.^17^ In our study, CT was performed in most patients with biliary trauma (77%), but each case required further diagnostic testing for confirmation of the injury. MRCP is a useful diagnostic tool in cases of biliary leaks.^18,19^ Aduna et al found that MRCP was 95% sensitive and 100% specific for biliary leaks in patients after cholecystectomy.^19^ However, MRCP may not always be feasible in the trauma setting given the high acuity of the patient. Additionally, associated injuries may complicate the clinical picture, and suspicion for biliary injury may not be high enough to pursue dedicated imaging.^3^ Despite the effectiveness of MRCP, the diagnosis of traumatic biliary leaks is usually made during exploratory laparotomy or after the development of late complications.^20,21^ Our study demonstrates this diagnostic dilemma as diagnosis was often delayed (median 7 days from injury), and MRCP was only performed prior to ERCP in 1 patient with biliary trauma. Of note, MRCP did not accurately diagnose the biliary leak in that case.

While small intrahepatic bile leaks may respond well to conservative treatment, larger bile leaks are prone to complications.^22^ Early treatment with percutaneous pigtail drainage of bilomas and successful ERCP can lead to resolution of bile leaks and prevent the infection of bilomas.^14,23-29^ Infected bilomas occurred twice in our population: in a patient who underwent ERCP 4 days after trauma and in a patient who underwent ERCP 18 days after trauma. Given the risks for infection and the previously documented successful management of bile leaks, we advocate for early ERCP in patients when clinical suspicion is high for a major bile leak, as well as interventional radiology–guided drainage of bilomas prior to ERCP.^14,23-29^ Our study supports this assertion, as ERCP was effective in our biliary trauma cohort with 100% of bile leaks resolved in the patients who underwent repeat ERCP and no significant morbidity or mortality secondary to the bile leak.

Traumatic injury to the pancreas is less common than biliary tract injury and may be more difficult to diagnose. Main pancreatic duct disruptions portend a worse prognosis than other pancreatic trauma complications, and key to the diagnostic evaluation of pancreatic trauma is the ability to evaluate the integrity of the main pancreatic duct.^9,30^ CT has demonstrated poor sensitivity for the evaluation of pancreatic duct injuries.^1,2,7^ Of the 12 patients with pancreatic trauma without biliary trauma in this cohort, 9 (75%) had pancreatic duct leaks found on ERCP. Each of those 9 was evaluated with CT prior to ERCP, and radiology interpreted the initial CT as concerning for pancreatic injury in 8 cases (89%), with concern for pancreatic duct injury specifically documented in 6 cases (67%).

MRCP has become the noninvasive imaging method of choice when evaluating for pancreatic duct injury; however, MRCP use in our cohort was fairly limited with only 2 of the 9 patients with ERCP-confirmed pancreatic duct leaks undergoing MRCP prior to ERCP. While prior studies have suggested MRCP to be effective at diagnosing pancreatic duct injuries,^1,9^ we cannot confirm this finding given the low sample size of our study and that MRCP was diagnostic in just 1 of the 2 patients with later confirmed duct leaks.

ERCP is the most accurate method for assessing the location and extent of pancreatic duct injury and can be performed prior to, during, or after surgical management.^31^ ERCP in stable patients who do not require emergent surgery allows for nonoperative treatment in the absence of ductal injury and earlier operative treatment or primary therapy such as stent placement in the presence of ductal injury.^7,32,33^ ERCP can also help to manage local complications of pancreatic trauma as both endoscopic transpapillary and transmural drainage can effectively manage pseudocysts and fistulas.^2,4,9,34^ In our cohort, repeat ERCP with pancreatic duct stenting led to resolution of the pancreatic duct leak in 57% of patients (4/7). ERCP was also generally well tolerated with no noted procedural adverse events in the pancreatic trauma cohort. Given the potential for morbidity related to surgical management, ERCP appears to be a reasonable management strategy for stable trauma patients with suspected pancreatic duct leaks. We agree with the necessity of surgery in unstable patients which was the case for numerous patients in this study.

Our study has some noteworthy limitations. The small sample size limits generalizability as does the retrospective nature of the study. Although we were assessing the efficacy of ERCP, numerous interventions, including percutaneous and surgical interventions, were used to manage the traumatic injuries in these patients, so determining the efficacy of one intervention by itself is difficult.

## CONCLUSION

ERCP was an effective diagnostic and therapeutic tool in this population of patients who sustained pancreaticobiliary injuries after abdominal trauma. Performing early ERCP in stable trauma patients with suspected pancreaticobiliary injuries may be warranted.

## Notes

*This article meets the Accreditation Council for Graduate Medical Education and the American Board of Medical Specialties Maintenance of Certification competencies for Patient Care and Medical Knowledge.*

## References

[1] Søreide K, Weiser TG, Parks RW. Clinical update on management of pancreatic trauma. HPB (Oxford). 2018;20(12):1099-1108. doi: 10.1016/j.hpb.2018.05.009 30005994

[2] Larsen M, Kozarek R. Management of pancreatic ductal leaks and fistulae. J Gastroenterol Hepatol. 2014;29(7):1360-1370. doi: 10.1111/jgh.12574 24650171

[3] Bajaj JS, Dua KS. The role of endoscopy in noniatrogenic injuries of the liver. Curr Gastroenterol Rep. 2007;9(2):147-150. doi: 10.1007/s11894-007-0009-0 17418060

[4] Thomson DA, Krige JE, Thomson SR, Bornman PC. The role of endoscopic retrograde pancreatography in pancreatic trauma: a critical appraisal of 48 patients treated at a tertiary institution. J Trauma Acute Care Surg. 2014;76(6):1362-1366. doi: 10.1097/TA.0000000000000227 24854301

[5] Pavlidis ET, Psarras K, Symeonidis NG, Geropoulos G, Pavlidis TE. Indications for the surgical management of pancreatic trauma: an update. World J Gastrointest Surg. 2022;14(6):538-543. doi: 10.4240/wjgs.v14.i6.538 35979422 PMC9258242

[6] Kim S, Kim JW, Jung PY, Diagnostic and therapeutic role of endoscopic retrograde pancreatography in the management of traumatic pancreatic duct injury patients: single center experience for 34 years. Int J Surg. 2017;42:152-157. doi: 10.1016/j.ijsu.2017.03.054 28343030

[7] Kim HS, Lee DK, Kim IW, The role of endoscopic retrograde pancreatography in the treatment of traumatic pancreatic duct injury. Gastrointest Endosc. 2001;54(1):49-55. doi: 10.1067/mge.2001.115733 11427841

[8] Jeroukhimov I, Zoarets I, Wiser I, Diagnostic use of endoscopic retrograde cholangiopancreatectography for pancreatic duct injury in trauma patients. Isr Med Assoc J. 2015;17(7):401-404. 26357712

[9] Bhasin DK, Rana SS, Rawal P. Endoscopic retrograde pancreatography in pancreatic trauma: need to break the mental barrier. J Gastroenterol Hepatol. 2009;24(5):720-728. doi: 10.1111/j.1440-1746.2009.05809.x 19383077

[10] Lin BC, Wong YC, Chen RJ, Major pancreatic duct continuity is the crucial determinant in the management of blunt pancreatic injury: a pancreatographic classification. Surg Endosc. 2017;31(10):4201-4210. doi: 10.1007/s00464-017-5478-0 28281124

[11] Lin BC, Chen RJ, Fang JF, Hsu YP, Kao YC, Kao JL. Management of blunt major pancreatic injury. J Trauma. 2004;56(4):774-778. doi: 10.1097/01.ta.0000087644.90727.df 15187740

[12] Lubezky N, Konikoff FM, Rosin D, Carmon E, Kluger Y, Ben-Haim M. Endoscopic sphincterotomy and temporary internal stenting for bile leaks following complex hepatic trauma. Br J Surg. 2006;93(1):78-81. doi: 10.1002/bjs.5195 16315338

[13] Rio-Tinto R, Canena J. Endoscopic treatment of post-cholecystectomy biliary leaks. GE Port J Gastroenterol. 2021;28(4):265-273. doi: 10.1159/000511527 34386554 PMC8314759

[14] Bridges A, Wilcox CM, Varadarajulu S. Endoscopic management of traumatic bile leaks. Gastrointest Endosc. 2007;65(7):1081-1085. doi: 10.1016/j.gie.2006.11.038 17531646

[15] Testoni PA. Endoscopic pancreatic duct stent placement for inflammatory pancreatic diseases. World J Gastroenterol. 2007;13(45):5971-5978. 18023085 10.3748/wjg.v13.45.5971 PMC4250876

[16] Sundaram S, Patra BR, Choksi D, Outcomes and predictors of response to endotherapy in pancreatic ductal disruptions with refractory internal and high-output external fistulae. Ann Hepatobiliary Pancreat Surg. 2022;26(4):347-354. doi: 10.14701/ahbps.22-002 35995583 PMC9721253

[17] LeBedis CA, Anderson SW, Mercier G, The utility of CT for predicting bile leaks in hepatic trauma. Emerg Radiol. 2015;22(2):101-107. doi: 10.1007/s10140-014-1262-9 25146931

[18] Vitellas KM, El-Dieb A, Vaswani KK, MR cholangiopancreatography in patients with primary sclerosing cholangitis: interobserver variability and comparison with endoscopic retrograde cholangiopancreatography. AJR Am J Roentgenol. 2002;179(2):399-407. doi: 10.2214/ajr.179.2.1790399 12130441

[19] Aduna M, Larena JA, Martín D, Martínez-Guereñu B, Aguirre I, Astigarraga E. Bile duct leaks after laparoscopic cholecystectomy: value of contrast-enhanced MRCP. Abdom Imaging. 2005;30(4):480-487. doi: 10.1007/s00261-004-0276-2 15688109

[20] Fleming KW, Lucey BC, Soto JA, Oates ME. Posttraumatic bile leaks: role of diagnostic imaging and impact on patient outcome. Emerg Radiol. 2006;12(3):103-107. doi: 10.1007/s10140-005-0453-9 16369810

[21] Molinari M. Traumatic bile duct injuries. In: Garbuzenko DV, ed. Actual Problems of Emergency Abdominal Surgery. IntechOpen; 2016. doi: 10.5772/64535

[22] Zakaria HM, Oteem A, Gaballa NK, Risk factors and management of different types of biliary injuries in blunt abdominal trauma: single-center retrospective cohort study. Ann Med Surg (Lond). 2020;52:36-43. doi: 10.1016/j.amsu.2020.02.009 32211187 PMC7082429

[23] Anand RJ, Ferrada PA, Darwin PE, Bochicchio GV, Scalea TM. Endoscopic retrograde cholangiopancreatography is an effective treatment for bile leak after severe liver trauma. J Trauma. 2011;71(2):480-485. doi: 10.1097/TA.0b013e3181efc270 21206287

[24] Sharma BC, Mishra SR, Kumar R, Sarin SK. Endoscopic management of bile leaks after blunt abdominal trauma. J Gastroenterol Hepatol. 2009;24(5):757-761. doi: 10.1111/j.1440-1746.2008.05703.x 19054254

[25] Hommes M, Kazemier G, Schep NW, Kuipers EJ, Schipper IB. Management of biliary complications following damage control surgery for liver trauma. Eur J Trauma Emerg Surg. 2013;39(5):511-516. doi: 10.1007/s00068-013-0304-4 26815449

[26] Bajaj JS, Spinelli KS, Dua KS. Postoperative management of noniatrogenic traumatic bile duct injuries: role of endoscopic retrograde cholangiopancreaticography. Surg Endosc. 2006;20(6):974-977. doi: 10.1007/s00464-005-0472-3 16738995

[27] Yuan KC, Wong YC, Fu CY, Chang CJ, Kang SC, Hsu YP. Screening and management of major bile leak after blunt liver trauma: a retrospective single center study. Scand J Trauma Resusc Emerg Med. 2014;22:26. doi: 10.1186/1757-7241-22-26 24735590 PMC4012546

[28] Dumonceau JM, Tringali A, Papanikolaou IS, Endoscopic biliary stenting: indications, choice of stents, and results: European Society of Gastrointestinal Endoscopy (ESGE) Clinical Guideline–Updated October 2017. Endoscopy. 2018;50(9):910-930. doi: 10.1055/a-0659-9864 30086596

[29] Gad EH, Zakaria H, Kamel Y, Surgical (open and laparoscopic) management of large difficult CBD stones after different sessions of endoscopic failure: a retrospective cohort study. Ann Med Surg (Lond). 2019;43:52-63. doi: 10.1016/j.amsu.2019.05.007 31198552 PMC6556483

[30] Biffl WL, Ball CG, Moore EE, Current use and utility of magnetic resonance cholangiopancreatography, endoscopic retrograde cholangiopancreatography, and pancreatic duct stents: a secondary analysis from the Western Trauma Association multicenter trials group on pancreatic injuries. J Trauma Acute Care Surg. 2023;95(5):719-725. doi: 10.1097/TA.0000000000003990 37125949

[31] Buccimazza I, Thomson SR, Anderson F, Naidoo NM, Clarke DL. Isolated main pancreatic duct injuries spectrum and management. Am J Surg. 2006;191(4):448-452. doi: 10.1016/j.amjsurg.2005.11.015 16531134

[32] Rogers SJ, Cello JP, Schecter WP. Endoscopic retrograde cholangiopancreatography in patients with pancreatic trauma. J Trauma. 2010;68(3):538-544. doi: 10.1097/TA.0b013e3181b5db7a 20016385

[33] Siiki A, Ahola R, Vaalavuo Y, Antila A, Laukkarinen J. Initial management of suspected biliary injury after laparoscopic cholecystectomy. World J Gastrointest Surg. 2023;15(4):592-599. doi: 10.4240/wjgs.v15.i4.592 37206082 PMC10190719

[34] Richey JS, Manning BM, Jones WB. Outcomes of endoscopic stenting for traumatic biliary and pancreatic fistulae. Am Surg. 2016;82(7):588-593. 27457856

